# Comparative *in vitro* evaluation of five *Commelina* forage species on ruminal fermentation and methanogenesis

**DOI:** 10.1016/j.heliyon.2023.e22769

**Published:** 2023-11-27

**Authors:** Kebede Gelgelo, Yisehak Kechero, Dereje Andualem

**Affiliations:** a Arba Minch University, College of Agricultural Sciences, Department of Animal Sciences, P O Box 21, Gamo Zone, Arba Mincch, Ethiopia; b Dilla University, College of Agriculture and Natural Resources, Department of Animal and Range Sciences, P O Box 419, Gedeo Zone, Dilla, Ethiopia

**Keywords:** Altitudinal dynamics, *Commelina* species, Gas production, Konso, Methane, Seasonal dynamics

## Abstract

*In vitro* gas measurement study was known to be a helpful tool for investigating the nutritional quality of feed for ruminant animals. This research was conducted to explore the ruminal fermentation and methane emission potential, and their variability pattern with changing seasons and altitudes for *Commelina* species, using in vitro *t*es*t.* Samples of the species were collected from different altitudes and seasons and used for the in vitro degradability study. Five species (*C.africana, C. albescence, C. benghalensis, C. imberbis* and *C. diffusa),* two altitudes (low and mid), and two seasons (wet and dry) were arranged in a 5 × 2 x 2 factorial manner in a completely randomized design, with three repeats of each treatment. *In vitro* gas and methane production as well as methane to total gas ratio were all meaningfully (P < 0.001) affected by season and altitude with the highest values observed in wet season and mid altitudes. At 24 h incubation, 44.76 and 37.82 ml/200 mg DM of total gas production was noted for wet and dry seasons respectively, while 39.77 and 47.38 ml/200 mg DM was recorded for low and mid altitudes respectively. Average gas production from immediately fermentable fractions (a) for wet season (4.21 ml) was reasonably (P < 0.001) higher than those for dry season (2.16 ml). Midlands (5.04 ml) had higher (P < 0.001) ‘a’ value than lowlands (2.77 ml). Both methane production and methane to total gas ratio exhibited a tangible decrease (P < 0.001) from wet season to dry season. 4.88 ml/200 mg DM and 6.52 ml/200 mg DM methane records were recorded for lowlands and midlands respectively. It appears that *Commelina* species contain nutrients that degrade in vitro, highlighting their potential supplement value for animals. The promising gas production potential added to its low methanogenic coefficient relative to the other species makes *C.* diffusa to the preferred supplement for poor-quality roughages followed by *C. benghalensis* and *C.* imberbis and the rest species being least preferred. But further nutrient analysis, minerals, secondary metabolites and the like, was required. And, in vivo trials must be conducted to strengthen the implications of this study.

## 1 Introduction

Non-conventional feeds (NCFs) were known to play a significant role in sustaining and supporting livestock production by low-income farmers. In the aforesaid context, their weighty role can be defined by their easy accessibility and their nutritional value [1,2]. Furthermore, non-conventional feeds were kinds of low-cost feeds that farmers at all levels of income could afford and thus contributed considerably to increasing the economic returns of livestock farming by increasing the diversity of feed resources and reducing the competition between humans and livestock for food crops, which is currently the sector's most bothersome issue [2]. In countries like Ethiopia, with large potential of non-conventional feeds available across various corners of the country to be utilized for all their livestock feed potencies [3], research activities concerned with non-conventional feeds deserve a lot of attention.

Examining the chemical composition of feed helps to obtain precise information on the quantitative aspect of the chemical constituents, both desirable and undesirable, of the feed materials [4]. The outputs of chemical analysis will be highly meaningful if they were supported by digestibility studies, because the high nutrient composition does not necessarily mean better nutritive value as the ingredients actually detected in the feed may not necessarily be digested and utilized by the animals due to digestibility and availability limiting factors in the feed materials themselves [5].

Gas production was one of the most commonly used in vitro feed quality assessment methods. It was a test tube simulation of microbial digestion actually happening in ruminants. Rumen microbes digest nutrients and make them available for ruminants in all forms of feed. Therefore, the in vitro gas measurement study was a helpful tool for investigating feed quality for ruminant animals. By quantifying the amount of gas produced during incubation, it is possible to predict the extent and rate of fermentation of both the soluble and insoluble fractions of feedstuffs [6,7]. *In vitro* gas production potential of a given forage species was reported to be influenced by many factors. Comparing different cacti varieties, Menezes et al. [8], reported that *Opuntica stricta* had a higher value for total gas production potential (186.54 ml/g DM) than *Pilosocereus gounelle* (72.66 ml/g DM). Due to advanced maturity and low soil moisture, plants had higher fiber fractions and lower soluble nutrients in the dry season as compared to wet season [9]. According to Asmamaw et al. [10], gas production at 24 h incubation, gas production from immediately fermentable fraction (a), gas production from insoluble but potentially fermentable fraction (b), potential gas production (a+b), and the rate constant of gas production (c) were significantly greater (P < 0.05) for *Prosopis juliflora* pods collected during wet season whereas, lag time (L) of *P. juliflora* pods were higher (P < 0.05) during the dry season. The tree legume *Albizia gummifera* growing at higher altitudinal regions in hot humid tropical climatic conditions of southwestern Ethiopia, was reported to have higher in vitro dry matter digestibility (405.2 ± 3.4 g/kg DM) than those of lower altitudinal regions (394.9 ± 2.0 g/kg DM) [11].

In the Konso Zone, southern Ethiopia, *Commelina* species were considered enormously asvaluable forage herb used by livestock keepers as feed for all kinds of livestock species. Livestock keepers in the study area allow *Commelina* species to grow as an alley crop on arable land. The species growing in harmony and socially with food crops provide plenty of multiple advantages for agricultural productivity in the area besides animal feed usage [12] Few studies conducted so far [13,14] confirmed and magnified the medicinal value of the herb. According to Lanyasunya et al. [15], *Commelina difusa* has 17.71 % CP, 36.08 NDF, 22.72 % ADF, 13.36 % hemicellulose and 20.50 % ash. On the other hand, *Commelina benghalensis* was reported to have CP, NDF, NDF and ADF values of 17.59 %, 32.60 %, 21.60 % and 11.00 % respectively [16]. Furthermore, El-Hamid and El Bous [17] pointed out that *Commelina* species have a potential to flourish well in different habitat types and tolerate environmental stress, reinforcing the feed use potential of the species under various ecological conditions. But, the multiple applicability of the species in feed sector and relative nutritional potentials of different species of the plant were still desirable. Therefore, this study hypothesizes that with changing seasons and altitudes, the in vitro gas and methane production of *Commelina* species could vary significantly. And thus, the aim of this research work was to explore the ruminal fermentation and methane production potential and examine their pattern of variability with changing seasons and altitudes using in vitro test.

## 2 Materials and methods

### 2.1 Description of the study area

The study was conducted in the Konso zone of southern Ethiopia.

**Fig. 1:**
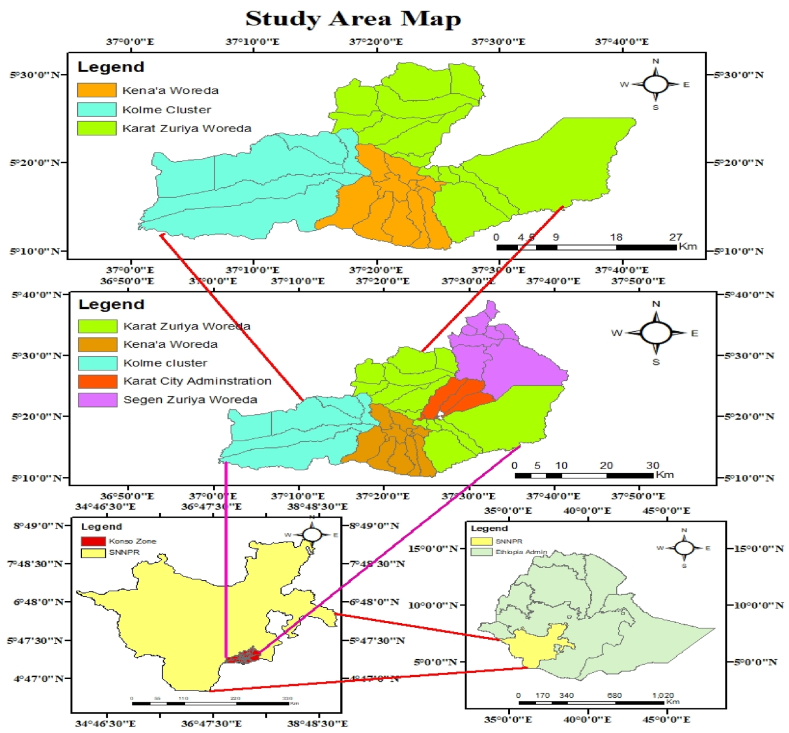
Map of the study area.

The study site is located at 5010′0″ to 5040′0″N latitude and 3700′0″ to 37045′0″ E longitude (Fig. 1). The total area of the Zone is 2016.24 Km^2^. The altitude of the area is between 501 and 2000 m above sea level. The main agro-ecological divisions of Konso are 70 % low altitude and 30 % accounting midlatitudes. The mean annual temperature of the zone ranges between 17.6 and 27.50 °C. The mean annual rainfall ranges between 601 and 1200 mm [18].

**Fig. 2:**
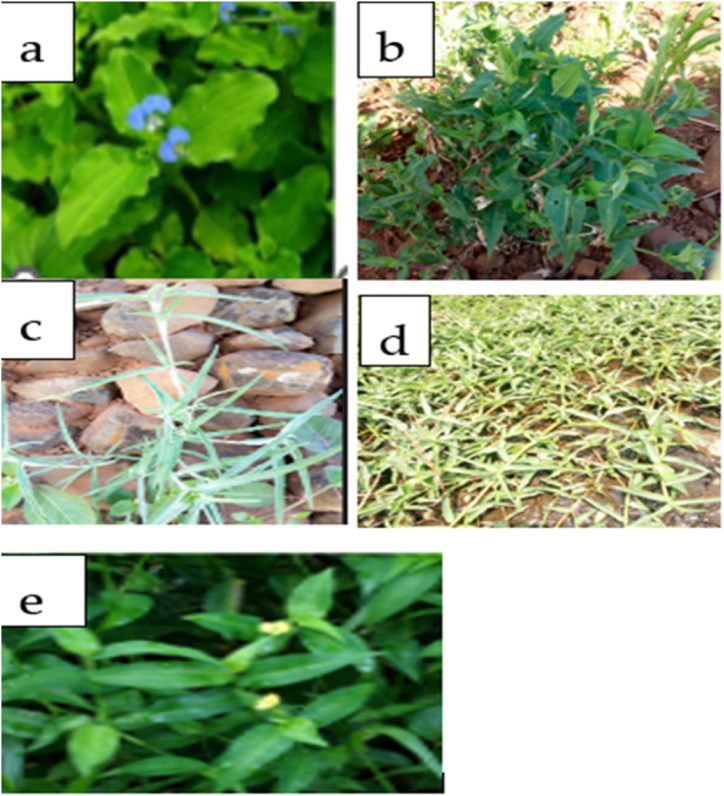
The five *Commelina* species under study: (a) *C. benghalensis*; (b) *C. imberbis*; (c) *C. al-bescense;* (d) *C. diffusa*; (e) *C. africana* (Adopted from Kebede et al., [12]).

The five *Commelina* species used in this study were depicted in Fig. 2. *C. imberbis* (b)*, C. diffusa*, (d) and *C. benghalensis* (a) were abundant at different altitudes, from lowlands to highlands. However, *C. albescens* (c) and *C. africana* (e) grow abundantly at altitudes ranging from 300 to 1700 masl, but it is difficult to find these species growing above 2000 masl [12,19].

### 2.2 Sample collection

Out of the five districts of Konso zone, three districts, Karat Zuriya district, Kena'a and Kolme (Fig. 1), were selected randomly. Based on the availability of the experimental plant, 15 villages (6 from mid altitude and 9 from low altitude to maintain altitudinal influence) were selected purposively, out of the 30 villages of the three districts. Samples of all available *Commelina* species were collected from all of the selected 15 villages, mixed thoroughly per altitude where three subsamples were taken per altitude for each species and brought to the animal nutrition laboratory for in vitro investigation. Dry season samples were collected in the middle of the dry period to balance the influence of prolonged water deficiency and extreme maturity, while wet season samples were collected in the middle of the flowering stage [20].

### 2.3 *In vitro* gas production

Rumen fluid was obtained from the rumen of three Adilo sheep breeds from Dilla University farm that were housed in individual cages. They were fed twice daily with a diet containing pasture hay mixture (60 %) and concentrate mixture (40 %) that had free access to clean drinking water and mineral licks. A sample of rumen content (about 1 L) was collected using a rumen tube before the morning meal in thermos flasks, taken immediately to the laboratory, strained through two layers of cheese cloth, and kept at 39 °C under a CO2 atmosphere. About 200 mg of dry sample (milled through a 1.0 mm sieve) was incubated in vitro with rumen fluid in a calibrated glass syringe of 100 ml in triplicate. Vaseline was applied to the pistons to ease movement and prevent the escape of gas. The syringes were pre-warmed at 39 °C before the addition of 30 ml of buffer mixture and rumen liquor into each syringe. The syringes were shaken for 30 min after incubation started and every hour for the first 10 h of incubation. Rhodes grass was used as a control and the same procedure was applied as for the feed samples. Blanks with buffered rumen fluid without feed samples were also included in triplicate. All syringes were incubated in a water bath maintained at 39 °C. Gas production was recorded after 3, 6, 12, 24, 48, and 72 h of incubation. The gas production characteristics were estimated by PROC NLIN of SAS [21] fitting to equation G = a + b (1 – e^-c (t-L)^) [22], where G is the gas production (ml/200 mg DM) at time t, a is gas production from the immediately soluble fraction (ml), b is gas production from the insoluble but degradable fraction (ml), a + b = Potential gas production (ml); c = the rate constant of gas production (fraction/h); L - lag time.

Post the incubation period, 4 ml of (10 M) sodium hydroxide (NaOH) was dispensed into each incubated sample. Sodium hydroxide was added to absorb carbon dioxide produced during fermentation and the remaining volume of gas was recorded as methane according to Fievez et al. [23]. The average of the volume of gas produced from the blanks was deducted from the volume of gas produced from samples. The methane concentration was calculated as Jayanegara et al. [24]:

Methane concentration = net methane production/net gas production

### 2.4 Experimental design and data analysis

A completely randomized design was used in a factorial arrangement (Five species, two altitudes, and two seasons) with three repetitions per treatment, as follows:

Yijk=μ+αi+βj+δk+αβij+αδik+Ɛijk;where:

Yijk = is the response variable; μ = overall mean; αi = species effect; βj = effect of the altitude (Low or Mid); δk = effect of the season (Dry or Wet); αβij = Effect of the species × altitude interaction; αδij = Effect of species × season interaction; Ɛijk = the random residual error.

The data were analyzed using the PROC GLM of SAS [21]. Means were separated using the Duncan Multiple Range Test. The level of significance was determined at (P < 0.05).

## 3 Results

### 3.1 Seasonal variability in *in vitro* gas and methane production of *commelina* species

Seasonal dynamics of in vitro gas and methane production of *Commelina* species was depicted in Table 1.

**Table 1:** Seasonal dynamics of *In vitro* Gas (ml/200 gm DM) and Methane (CH4) production of *Commelina* species.

		Incubation Period						CH4	CH4/Total Gas (v:v)
Season	Species	3	6	12	24	48	72		
Wet season	*C.benghalensis*	8.05^a^	13.05^bc^	19.80^bc^	45.00^bc^	53.30^c^	80.00^bc^	7.25^abc^	0.091^ab^
	*C.imberbis*	8.15^a^	14.25^b^	20.70^ab^	46.80^b^	52.15^c^	77.28^c^	6.50^bc^	0.084^b^
	*C.diffusa*	9.50^a^	16.50^a^	24.50^a^	51.00^a^	58.00^b^	72.50^d^	5.80^c^	0.079^b^
	*C.africana*	7.00^a^	12.00^cd^	15.05^d^	42.00^cd^	64.00^a^	83.85^a^	8.98^a^	0.11^a^
	*C.albescence*	7.50^a^	11.50^d^	16.30^cd^	39.00^d^	58.50^b^	81.60^ab^	7.88^ab^	0.096^ab^
	Seasonal Mean	8.04^A^	13.46^A^	19.27^A^	44.76^A^	57.19^A^	79.05^A^	7.28^A^	0.092^A^
Dry Season	*C.benghalensis*	5.50^a^	10.08^b^	14.50^b^	38.00^ab^	46.75^b^	71.50^b^	5.55^bc^	0.078^ab^
	*C.imberbis*	6.00^a^	11.15^ab^	16.00^a^	40.53^a^	45.50^b^	70.50^b^	5.23^c^	0.074^b^
	*C.diffusa*	6.50^a^	12.60^a^	16.50^a^	40.50^a^	47.00^b^	65.05^c^	3.65^d^	0.056^c^
	*C.africana*	5.00^a^	8.00^c^	11.00^c^	34.00^b^	54.50^a^	76.85^a^	6.33^a^	0.082^a^
	*C.albescence*	5.04^a^	8.50^c^	14.00^b^	36.05^ab^	53.15^a^	73.00^b^	5.83^ab^	0.079^a^
	Seasonal Mean	5.61^B^	10.07^B^	14.40^B^	37.82^B^	49.38^B^	71.38^B^	5.32^B^	0.074^B^
Significance	SEM	0.37	0.57	0.87	1.14	1.31	1.27	0.33	0.0031
	Season	0.001	<0.0001	<0.0001	<0.0001	<0.0001	<0.0001	<0.0001	<0.0001
	Species	0.19	<0.0001	<0.0001	<0.0001	<0.0001	<0.0001	<0.0001	0.0002
	Species x season	0.97	0.42	0.029	0.059	0.102	0.66	0.33	0.18

^a,b,c,d^Column means with different superscripts shows significant between-species difference (P < 0.05); ^A,B^Column means with different superscripts shows significant seasonal difference; SEM=Standard Error of the Mean.

In vitro gas and methane production as well as methane to total gas ratio were all meaningfully (P < 0.001) affected by season with the highest observation in the wet season compared to the dry season. Similarly, between species variability was also significant for all parameters examined (P < 0.001) except gas production at 3 h. On the other hand, the species × season interaction effect was significant for none of the parameters examined in this study.

**Fig. 3:**
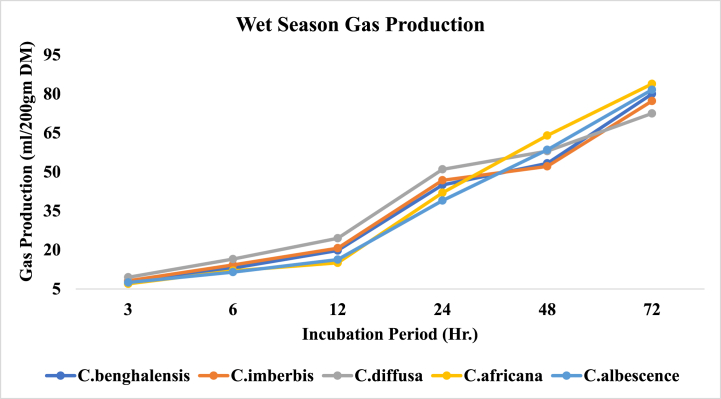
*In vitro* gas production of *Commelina* species in wet season.

Wet season observation indicates that the leading potency of gas production throughout the incubation period is consistent for none of the Commelina species. There was no meaningful difference in the volume of gas produced by all of the species at the first observation of this study (3 h incubation) (P > 0.05). A tangible difference among the species in the gas production potential was primarily recorded at 6 h where C. diffusa scored the highest value (16.50 ml) (P < 0.001) and C. albescence produced the lowest gas (11.50 ml). C. diffusa persisted to generate the highest significant volume of gas (P < 0.001) till 24 h of incubation (51.00 ml). Where C. albescence also maintained its least gas releasing potential (39.00 ml). Though it had a comparable record with C. africana (42.00 ml). In the late periods of incubation, post 24 h, gas production potency was reversed so that C. africana became the most potent (P < 0.001) at 48 h (64.00 ml) and 72 h (83.85 ml) having C. albescence with it at 72 h (81.60 ml), whereas C. diffusa turned out to be the least gas emitting species with 58.00 ml and 72.50 ml of gas volumes recorded for it at 48 h and 72 h post incubation respectively. On the other hand, C. imberbis and C. benghalensis were consistently observed to have intermediate and analogous gas volumes at both late and early periods of incubation. Fig. 3 shows the in vitro gas production of Commelina species in the wet season.

**Fig. 4:**
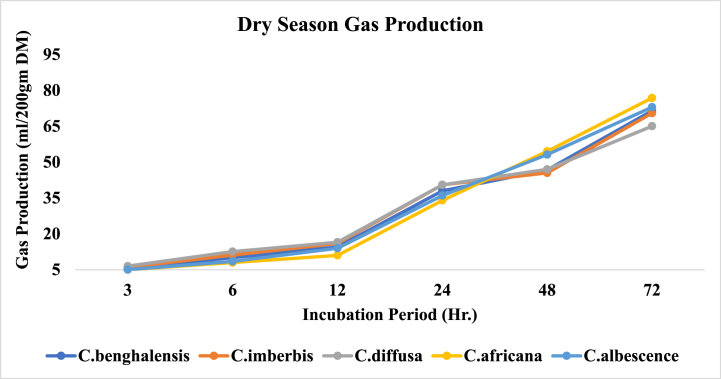
*In vitro* gas production of *Commelina* species in dry season.

In the dry season, as it had been for the wet season, all of the Commelina species produced equivalent (P > 0.05) volumes of gas 3 h post incubation. Similarly, 24 h gas production was also comparable among all species except *C. africana*, having lower records (34.00 ml) than C. diffusa *(40.*50 ml*)* and C. imberbis (*40.*53 ml). At 48 h of incubation, C. africana (54.50 ml) and C. albescence (53.15 ml) had the highest gas volumes over all the rest species which had the least comparable gas volumes. Based on the dry season gas volumes at 72 h, all Commelina species fall into three distinctive categories, C. africana as the top gas producer (76.85 ml) (P < 0.001), C. diffusa being the least (65.05 ml) and the remaining species having intermediate and analogous gas producing potential. Dry season gas production trends of Commelina species over the incubation periods are illustrated in Fig. 4.

Methane production potential of the *Commelina* species in the present study was in the range of 5.80–8.98 ml/200 gm DM in wet season which was dropped to 3.65–6.33 ml/200 gm DM in dry season. The highest wet season methane records were noted for C. africana (8.98 ml) and the lowest was observed in C. diffusa *(*5.80 ml) though it had comparable values with those of C. imberbis *(*6.50 ml) and C. benghalensis *(*7.25 ml). In the same way, the highest dry season methane values were congruently noted for both C. africana (6.33 ml) and C. albescence (5.83 ml) whereas C. diffusa (3.65 ml) continued to be the least methane producing species of all in the dry season as well. As it had been for methane production, the ratio of methane to total gas volume also exhibited a reasonable decrease (P < 0.001) from 0.092 in wet season to 0.074 in dry season.

### 3.2 Altitudinal variability in *in vitro* gas and methane production of *commelina* species

The altitudinal dynamics of in vitro gas and methane production by *Commelina* species under study were depicted in Table 2. *In vitro* gas and methane production as well as methane to total gas ratio were all meaningfully (P < 0.001) affected by altitudinal changes with the highest observation at the mid altitudes compared to low altitudes. Similarly, between species variability was also significant for all of the parameters examined (P < 0.001) except gas production at 3 h and 48 h (P > 0.05). On the other hand, the species × season interaction effect was insignificant for all of the parameters examined in this study (P > 0.05) except for gas production at 24 h and methane to total gas volume ratio (P < 0.05).

**Table 2:** Altitudinal dynamics of *In Vitro* Gas (ml/200 gm DM) and Methane production of *Commelina* species.

		Incubation Period						CH4	CH4/Total Gas (v:v)
Altitude	Species	3	6	12	24	48	72		
Low Altitude	*C.benghalensis*	5.00^a^	10.50^a^	14.80^a^	38.00^b^	46.75^a^	71.50^a^	5.55^a^	0.078^a^
	*C.imberbis*	6.15^a^	11.00^a^	15.70^a^	40.80^a^	45.50^a^	71.00^a^	5.45^a^	0.077^a^
	*C.diffusa*	6.50^a^	12.60^a^	16.00^a^	40.50^a^	47.00^a^	65.00^b^	3.65^b^	0.056^b^
	Altitudinal Mean	5.88^B^	11.37^B^	15.50^B^	39.77^B^	46.42^B^	69.17^B^	4.88^B^	0.070^B^
Mid Altitude	*C.benghalensis*	8.55^a^	13.35^c^	19.50^b^	45.00^b^	53.40^a^	80.00^a^	7.25^a^	0.091^a^
	*C.imberbis*	8.00^a^	14.40^b^	20.50^b^	46.15^b^	52.15^a^	77.15^ab^	6.50^a^	0.084^a^
	*C.diffusa*	9.50^a^	16.50^a^	24.50^a^	51.00^a^	58.00^a^	72.50^b^	5.80^a^	0.079^a^
	Altitudinal Mean	8.68^A^	14.75^A^	21.50^A^	47.38^A^	54.52^A^	76.55^A^	6.52^A^	0.085^A^
Significance	SEM	0.52	0.64	1.06	1.32	1.42	1.46	0.34	0.0033
	Altitude	0.0039	0.0011	0.0002	<0.0001	0.0015	<0.0001	0.0002	0.0002
	Species	0.26	0.019	0.021	0.0057	0.15	0.0007	0.0011	0.0009
	Species x Altitude	0.49	0.71	0.082	0.039	0.36	0.39	0.11	0.020

^^a,b,^c^column means with different superscripts shows significant between-species difference (P < 0.05); ^A,B^Column means with different superscripts shows significant altitudinal difference; SEM=Standard Error of the Mean.

**Fig. 5:**
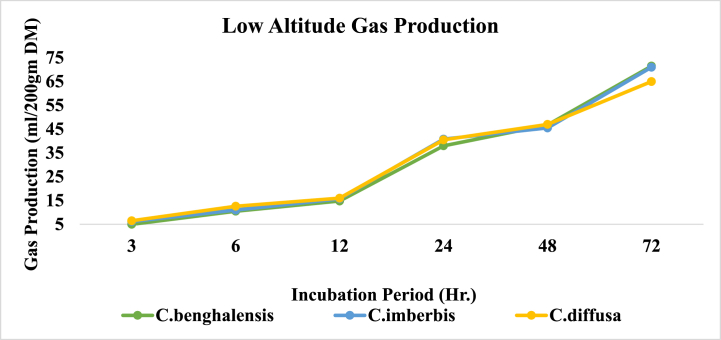
*In vitro* gas production of Commelina species in low altitude.

Trends in *in vitro* gas production potential of the *Commelina* species over the incubation hours in low-altitude are indicated by Fig. 5.

Lowland records of in vitro gas production for Commelina species indicate that, in the first 12 h of incubation, all of the species had equivalent gas producing potential (P > 0.05). The amount, in volume, of gas produced by C. benghalensis at 24 h (38.00 ml) was reasonably lower (P < 0.01) than that of the rest species which had the highest analogous records. On the other hand, C. diffusa had the least significant gas volume (P < 0.001) at 72 h (65.00 ml) than the other species which had the highest values comparable among each other at that moment.

**Fig. 6:**
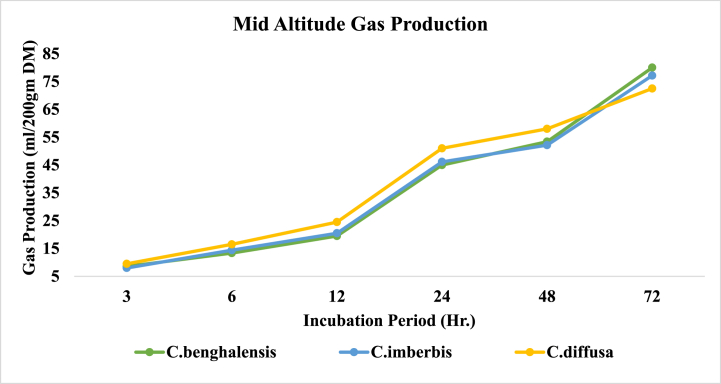
*In vitro* gas production of *Commelina* species in Mid Altitude.

In the mid altitudes, gas production (ml/200 gm DM) at the early 6 h significantly varies between the species in the way that the gas volume of C. diffusa (16.50) > C. imberbis (14.40) > C. benghalensis (13.35) (P < 0.001). However, the gas production of C. diffusa after 24 h of incubation (51.00 ml) was considerably higher than those of the remaining species which had similar gas volumes records. In general, total gas production at 72 h by C. benghalensis (80.00 ml) was equivalent to C. imberbis (77.15 ml) but reasonably higher (P < 0.001) than that of C. diffusa *(72.*50 ml*).* At mid altitudes, C. diffusa gas production was rapid over all the other species post 3 h till 48 h, from when its potential falls relative to the rest species (Fig. 6).

With advancing altitudes, a significant increment pattern was noted for methane production potential and methane to total gas ratio of *Commelina* species. On average 4.88 ml/200 mg DM and 6.52 ml/200 mg DM methane records were recorded for lowlands and midlands respectively. Midland observations indicate that all of the species had equivalent methane production potential and methane to total gas ratio. But in the lowlands, the methane production potential significantly varying between species (P < 0.001) in such a way that, the records of C. benghalensis (5.55 ml) = C. imberbis (5.45 ml) > C. diffusa *(3.*65 ml*)*. Correspondingly, the methane to total gas ratio in the lowlands showed a significant difference among the species where *C. benghalensis* (0.078) and *C. imberbis* (0.077) had comparable values higher than C. diffusa *(0.056)*.

### 3.3 Seasonal variability in *in vitro* gas production characteristics of *commelina* species

Seasonal dynamics of in vitro gas production characteristics of *Commelina* species are depicted in Table 3. All of the in vitro gas production characteristics were significantly (P < 0.001) affected by season. Gas production from immediately soluble fractions (a) and the rate of gas production (c) were both higher in the wet season as compared to dry season. Whereas, gas production from insoluble but potentially degradable fractions (b), potential gas production (a+b) and lag time were all higher in dry season as compared to wet season observations. On the other hand, the species × season interaction effect was significant for all of the gas production parameters except gas production from immediately soluble fractions.

**Table 3:** Seasonal dynamics of *In Vitro* Gas production characteristics of *Commelina* species.

		Gas production characteristics				
**Season**	**Species**	a	b	c	a+b	LT
Wet season	*C.benghalensis*	4.28^b^	94.80^c^	0.029^c^	99.08^c^	1.05^c^
	*C.imberbis*	4.77^ab^	89.83^d^	0.039^b^	94.59^d^	0.71^d^
	*C.diffusa*	5.99^a^	83.53^e^	0.055^a^	89.53^e^	0.63^d^
	*C.africana*	2.45^c^	112.77^a^	0.019^d^	115.22^a^	2.10^a^
	*C.albescence*	3.54^bc^	104.82^b^	0.014^d^	108.36^b^	1.55^b^
	Seasonal Mean	4.21^A^	97.15^B^	0.032^A^	101.36^B^	1.21^B^
Dry Season	*C.benghalensis*	2.11^c^	106.08^c^	0.020^c^	108.19^c^	1.36^c^
	*C.imberbis*	2.73^b^	111.99^b^	0.029^b^	114.72^b^	1.01^d^
	*C.diffusa*	3.40^a^	96.76^d^	0.039^a^	100.16^d^	0.97^d^
	*C.africana*	1.19^d^	121.91^a^	0.014^d^	123.10^a^	2.48^a^
	*C.albescence*	1.39^d^	114.10^b^	0.010^d^	115.49^b^	2.11^b^
	Seasonal Mean	2.16^B^	110.17^A^	0.022^B^	112.33^A^	1.59^A^
Significance	SEM	0.34	2.65	0.0031	2.34	0.14
	Season	<0.0001	<0.0001	<0.0001	<0.0001	<0.0001
	Species	<0.0001	<0.0001	<0.0001	<0.0001	<0.0001
	Species x season	0.29	0.0008	0.025	0.0015	0.0006

^a,b,c,d,e^Column means with different superscripts shows significant between-species difference (P < 0.05); ^A,B^Column means with different superscripts shows significant seasonal difference; a = Gas production from the immediately soluble fraction (ml), b = Gas production from the in-soluble but degradable fraction (ml), a + b = Potential gas production (ml); c = the rate constant of gas production (fraction/h); LT - lag time; SEM=Standard Error of the Mean.

Wet season records of gas production from immediately soluble fractions in *Commelina* species were in the range of 2.45–5.99 ml with a pooled average value of 4.21 ml. The highest and lowest ‘a’ values were observed in C. diffusa *(*5.99 ml) and C. africana (2.45 ml) respectively. In the dry season, C. diffusa continued to score the highest value of gas production from immediately soluble fractions (3.40 ml) whereas C. africana (1.19 ml) and C. albescence (1.19 ml) had the least values comparable with each other. Gas production from the insoluble but potentially degradable fractions as well as potential gas production were both markedly increased (P < 0.001) with advancement towards dry periods of the year. Wet season values of ‘b’ vary among species in the way that the records of C. africana (112.77 ml) > C. albescence (104.82 ml) > C. benghalensis (94.80 ml) > C. imberbis (89.83 ml) > C. diffusa (83.53 ml) (P < 0.001). Correspondingly, the highest and lowest potential gas production records in the wet season were noted for C. africana (115.22 ml) and C. diffusa (89.53 ml) respectively. Similarly, the highest (P < 0.001) dry season records of gas production from the insoluble but potentially degradable fractions as well as potential gas production were observed for C. africana whereas C. diffusa had the least significant score for both parameters.

In the present study, the rate constant of gas production by the *Commelina* species reasonably decreased (P < 0.001) from 0.032 ml in the wet season to 0.022 ml in the dry season. The parameter exhibited a consistent pattern of variability among species at both seasons of the year such that the records of C. diffusa > C. imberbis > C. benghalensis > C. albescence = C. africana. On average, the fermentation process lag time was 1.21 h in the wet season and augmented (P < 0.001) to 1.59 h in the dry season. In the wet season, the longest significant lag time was noted for C. africana (2.10 h) followed by C. albescence (1.55 h) and C. benghalensis (1.05 h) while C. diffusa (0.63 h) and C. imberbis (0.71 h) had the shortest comparable lag time records. This pattern of among species variability observed in the wet season was also consistently reflected in the dry season.

### 3.4 Altitudinal variability in *in vitro* gas production characteristics of *commelina* species

Table 4 shows the altitudinal dynamics of in vitro gas production characteristics of Commelina species. All of the in vitro gas production characteristics were significantly (P < 0.001) affected by altitude. Gas production from immediately soluble fractions (a) and the rate of gas production (c) were both higher at mid altitude as compared to their low altitude observations. While gas production from insoluble but potentially degradable fractions (b), potential gas production (a+b) and lag time were all higher at low altitudes than mid altitude observations. On the other hand, the species × season interaction effect was significant (P < 0.01) for insoluble but potentially degradable fractions and potential gas production but insignificant for all of the rest gas production parameters (P > 0.05).

**Table 4:** Altitudinal dynamics of *In Vitro* Gas production characteristics of *Commelina* species.

		Gas production characteristics				
Altitude	Species	A	b	c	a+b	LT
**Low Altitude**	*C.benghalensis*	2.15^b^	105.97^a^	0.021^b^	108.12^b^	1.38^a^
	*C.imberbis*	2.72^ab^	112.05^a^	0.030^b^	114.78^a^	1.04^b^
	*C.diffusa*	3.43^a^	95.61^b^	0.041^a^	99.04^c^	0.96^c^
	Altitudinal Mean	2.77^B^	104.54^A^	0.031^B^	107.31^A^	1.13^A^
**Mid Altitude**	*C.benghalensis*	4.28^a^	94.77^a^	0.031^b^	99.04^a^	1.02^a^
	*C.imberbis*	4.80^a^	90.31^b^	0.041^b^	95.12^a^	0.70^b^
	*C.diffusa*	6.04^a^	83.33^c^	0.056^a^	89.37^b^	0.64^c^
	Altitudinal Mean	5.04^A^	89.47^B^	0.042^A^	94.51^B^	0.79^B^
**Significance**	SEM	0.40	2.89	0.0033	2.53	0.074
	Altitude	0.0002	<0.0001	0.0003	<0.0001	<0.0001
	Species	0.0087	<0.0001	<0.0001	0.0001	<0.0001
	Species x Altitude	0.63	0.0036	0.25	0.0032	0.34

^a,b,c^ means with different superscripts shows significant between-species difference (P < 0.05); ^A,B^Column means with different superscripts shows significant altitudinal difference; a = Gas production from the immediately soluble fraction (ml), b = Gas production from the insoluble but degradable fraction (ml), a + b = Potential gas production (ml); c = the rate constant of gas production (fraction/h); LT - lag time; SEM=Standard Error of the Mean.

In the present study, gas production from immediately soluble fractions was in the range of 2.15–3.43 ml at low altitude. However, it raised up to 4.28–6.04 ml at mid-altitude. At mid-altitude, regardless of numerical variations, all species showed comparable gas production from immediately soluble fractions (P > 0.05). However, low altitude fractions of the parameter (a) noted for C. diffusa *(*3.43 ml) were reasonably higher (P < 0.01) than that of C. benghalensis (2.15 ml) while C. imberbis (2.72 ml) records were comparable with those of both species. At low altitude, the lowest significant value (P < 0.001) of gas production from the insoluble but potentially degradable fractions was noted for C. diffusa (95.61 ml) while the rest species had the highest corresponding values of the parameter (b). On the other hand, midland observations of ‘b’ values significantly vary (P < 0.001) between species in such a way that the records of C. benghalensis (94.77 ml) > C. imberbis (90.31 ml) > C. diffusa *(*83.33 ml).

As per the observations of the present study, the gas production rate constant of the Commelina species revealed a significant increasing trend (P < 0.001) as one moves up from lowlands (0.031 ml) to midlands (0.041 ml). But at both altitudes between species variability of gas production rate constant exhibited a uniform pattern, with C. diffusa having the greatest value (P < 0.001) and the rest species having the least comparable records of the stated parameter. On the other hand, lowland potential gas production values were highest for C. imberbis *(*114.78 ml); least for C. diffusa (99.04 ml) and intermediate for C. benghalensis *(*108.12 ml). In the midlands, the lowest significant potential gas production was noted for C. diffusa (89.37 ml) and the remaining species had highest (P < 0.001) comparable values. Findings of the present study also qualified that, the lag time of the fermentation process significantly decreased (P < 0.001) progressing from lowlands (1.13 h) to midlands (0.79 h). But with changing altitudes the length of lag time revealed a uniform tendency of variability among the species that the lag time of C. benghalensis > C. imberbis > C. diffusa.

## 4 Discussion

### 4.1 Seasonal variability in *in vitro* gas and methane production and gas production characteristics

The significant variability in *in vitro* gas production kinetics of *Commelina* species with varying seasons observed in the present study coincides with the findings of Tsegaye et al. [25] and was an indicator of the expected discrepancy in digestibility and nutritional quality of the plant species at different seasons of the year. The dynamics of in vitro gas production was an indicator of fermentability of readily digestible and slowly digestible components of feeds [6,7]. Immediately fermentable fractions of a feed had rapid initial gas production, declining at subsequent incubations [26]. So, the highest significant gas production noted for C. diffusa at earlier incubation hours might be an indicator of its better soluble nutrients’ composition. On the contrary, the relatively higher gas production values recorded for C. africana and C. albescence towards the late fermentation hours in the present study might be an indicator of higher and probably comparable fractions of slowly fermentable nutrients. Similarly, the intermediate and analogous gas volumes noted for C. imberbis and C. benghalensis consistently across the incubation process could be an indicator of their medium composition of both rapidly and slowly fermentable nutrients relative to the rest *Commelina* species examined.

The significant differences observed for the in vitro gas production parameters between seasons was an indicator of the existence of visible nutritional variability among *Commelina* species at differing seasons of the year in the study area. As to the implications of the present study, those species that have the highest immediately fermentable fraction would have the fastest rate of gas production constant as well as the shortest possible lag time. And in reverse, those species that have the highest insoluble but timely fermentable fraction would have the slowest rate of gas production constant and the longest possible lag time. In line with this, Tolera and Sundstøl [27], confirmed that poorly digestible feed components require plenty of exposure time to be hydrated and colonized by rumen microorganisms before they enter the fermentation process. Gas production from insoluble but timely degradable fractions of *Commelina* species in dry season was by far higher than those of wet season. It is possible that this could result in higher potential gas production (a+b) during the dry season.

Getachew et al. [28] suggested that the rate of nutrient fermentation in a particular feed sample is influenced by the microbial mass and the convenience of the feed material for microbial digestion. Besides, the value of gas production constant (c) was suggested to be equivalent to feed intake and therefore expected to affect the rate of ingesta passage along the gut, while potential gas production (a + b) was a correspondent of feed degradability [29]. To this end, depending on wet season observations of gas production constant, it follows that animals consuming C diffusa would have better feed intake followed by those consuming C imberbis and C. benghalensis while comparatively lower intake could be expected for animals relaying on C. africana and C. albescence-*based* diets. The relatively higher values of gas production from immediately fermentable fractions and the fastest gas production rate constant added to its shortest lag time in dry season as well make C diffusa a preferable dry season supplement over the other species followed by C imberbis and C. benghalensis.

The higher record of methane production in wet season as compared to dry season and the significant between-species variability observed for *Commelina* species in the present study were in agreement with the report of Tsegaye et al. [25], which could be the reflection of the influence of season on the nutritive potential of fodder species reported earlier [30]. According to Dereje et al. [31], there exists a strong and positive correlation between total gas production and methane potential of forage herbs. So, the leading potency of C. africana in methane production consistently observed at both seasons of the year in the present study could be explained by its highest total gas production during the late periods of fermentation. On the other hand, methane production represents the efficiency of microbial fermentation in the rumen and is associated with high contents of structural carbohydrates [24]. So, the least potency of C diffusa *in methane production could imply its lower content of the poorly digestible carbohydrate fractions. Thus, relatively more efficient microbial fermentation could be expected in line with* C diffusa *based livestock feeding compared to the rest* Commelina *species examined in the present study.*

#### 4.1.1 Altitudinal variability in *in vitro* gas and methane production and gas production characteristics

The advanced gas production potential of Commelina species at mid-altitude (8.68 ml at 3 h to 76.55 ml at 72 h) as compared to their low-altitude values (5.88 ml at 3 h to 69.17 ml at 72 h) was an indicator of better nutritional composition of the plants at mid-altitude than at low-latitude. This could be attributed to changes in environmental conditions like increased precipitation and declining temperature with advancing altitude, both contributing to a higher composition of soluble nutrients and reduced composition of the less digestible fractions in forage species [32]. In support of the present study's observations, Capelin et al. [33] reported that the concentration of soluble nutrients in a morphological fraction of plants increased with increasing altitude. In contrast to this, Qiao et al. [34] and Nathalie et al. [35] proposed that the nutritional quality of tropical trees, shrubs, and herbs decreased markedly with increasing altitude. The observed disagreements among the findings could be attributed to variation in potential of the areas associated environmental factors, edaphic properties influencing nutrient status, grazing pressure of the areas and management aspects of the grasslands [36].

The analog of gas production from immediately fermentable fractions and gas production rate constants of C. benghalensis and C. imberbis at both altitudes of the study area may indicate their equipotential to serve as supplements to low-quality diets in the study area next to C. diffusa. The higher records of gas production from insoluble but timely degradable fractions noted for Commelina species at low altitude than mid altitude could be qualified by the declining tendency in slow-digestible carbohydrate fractions of herbaceous fodder species with advancing altitudes reported earlier [37]. According to Boufennara et al. [32], the reduced environmental temperature and increased precipitation noted with advancing altitudes leads to decreased concentration of moderately fermentable fractions at higher altitude growing fodder species. Correspondingly, the longest lag time of fermentation was observed for low altitude could be due to the fermenting microbes requiring a reasonable amount of time to multiply and hydrate the slowly fermentable feed particles [27]. As to the reflections of McDonald et al. [5], the interwoven matrix of polymers in the slowly fermentable feed components creates barriers against the microbial invasion and limits their access to digesting enzymes of microbes thereby decreasing their rate of fermentation and lengthening their lag time in in vitro gas production.

Undoubtedly, high methane production implies substantial energy loss for animals and greenhouse gas emissions to the environment [38]. The ratio of methane to total gas production which is an indicator of methanogenic properties of a given fodder species per unit of degraded nutrient, basically serves as an objective criterion for screening out forage species for ruminant nutrition [39] considering both the nutritional and environmental implications of CH4 emissions. A low value of this ratio means a low methane producing potential of the most soluble fractions of a feed material, implying less methane production per unit of net gas volume production [38]. In view of this, midland observations reflect an analogy among all species. In contrast to the rest of the species with comparable potential, *C. diffusa* exhibits the least amount of methane production (3.65 ml/200 mg DM) and methanogenic coefficient (0.056) at low altitudes, making it the most preferable supplement.

The methane production potential of commelina species noted for midlands in the present study (6.52 ml/200 gm DM) was nearly consistent with the methane values noted for flower and leaf fractions of Stinging Nettle (Urtica simensis) (6.6 ml/200 mg DM) in the midlands of southern Ethiopia [31]. The same authors also reported a methane to total gas ratio of stem fractions and whole plant in the range 0.071–0.086, which nearly coincides with the midland observations of the present study (0.079–0.091). On the contrary, methane production (11.45 ml/200 mg DM) and methanogenetic coefficient (0.18–0.41), by far higher than those of the present study were reported for leguminous fodder species in the mild altitudes of Sidama region, southern Ethiopia [25]. The genetic potentials and habitat related factors like physical and chemical properties of soil influencing its nutritional status and thus the nutritional profile of the fodder crop growing on it could be the suggestible reasons for the divergence among the findings [36].

## 5 Conclusions

Gas volumes measured in in vitro experiments are related to the actual extent and rate of substrate degradation in vivo. So, the in vitro assessment of the Commelina species in the present study confirms that the herb species contain potentially degradable nutrients. This highlights the importance of these plants as potential supplements to poor quality diets for animals reared in Commelina growing areas. In vitro gas and methane production as well as methane to total gas ratio were all meaningfully (P < 0.001) affected by season and altitude with the highest values observed in wet season and mid altitudes. Among the Commelina species investigated, C. diffusa exhibited superior gas production at early hours of incubation emitting up to 51.00 ml of gas at 24 h in wet season with reduced potency post 24 h, highest significant gas production from immediately fermentable fractions (5.99 ml in wet and 3.40 ml in dry seasons) and fastest rate of gas production constant consistently across both seasons of the year. C. diffusa also had a lower methanogenic coefficient in the lowlands (0.056 ml). On the other hand, C. benghalensis and C. imberbis had analogous intermediate gas production potentials consistently at both early and late periods of the in vitro fermentation and the values of gas production from immediately fermentable fractions were comparable to that of C. diffusa. The promising gas production potential added to its low methanogenic coefficient relative to the other species makes C. diffusa the most ideal supplement to poor-quality roughages followed by C. benghalensis and C. imberbis, the rest species being the least preferred ones. But, further nutrient analysis, mineral, secondary metabolites and the like, was required. And, in vivo trials must be conducted to strengthen the implications of this study.

## Ethical statement

The animal study was reviewed and approved by Arba Minch University Animal Research Ethics Review Committee (Approval Number AMU/AREC/11/2015).

## Funding statement

This research did not receive any specific grant from funding agencies in the public, commercial, or not-for-profit sectors.

## Data availability statement

The data that support the findings of this study are available from the corresponding author on reasonable request.

## Additional information

No additional information is available for this paper.

## CRediT authorship contribution statement

**Kebede Gelgelo:** Conceptualization, Data curation, Investigation, Methodology, Writing - original draft. **Yisehak Kechero:** Methodology, Supervision, Writing - review & editing. **Dereje Andualem:** Methodology, Supervision, Writing - review & editing.

## Declaration of competing interest

The authors declare no conflict of interest.
